# *Notes from the Field:* Response to Measles Among Persons Evacuated from Afghanistan — Joint Base McGuire-Dix-Lakehurst, New Jersey, August–October 2021

**DOI:** 10.15585/mmwr.mm7117a3

**Published:** 2022-04-29

**Authors:** Nikki Pritchard, Mary Claire Worrell, Andrea Shahum, Angel Nwankwo, Dallas Smith, John J. Koch, Timothy Ballard

**Affiliations:** ^1^Navy Medicine Readiness and Training Command Twentynine Palms, Twentynine Palms, California; ^2^Center for Global Health, CDC; ^3^International SOS, Houston, Texas; ^4^60th Aeromedical Evacuation Squadron, Travis Air Force Base, California; ^5^Epidemic Intelligence Service, CDC; ^6^Navy Medicine Readiness and Training Command Camp Pendleton, Oceanside, California; ^7^Air Force Medical Readiness Agency, Falls Church, Virginia.

On August 29, 2021, the U.S. government initiated Operation Allies Welcome (OAW) to resettle eligible persons from Afghanistan. Evacuees were housed at military bases in the United States while completing immigration resettlement processing. On September 4, 2021, the Fort McCoy, Wisconsin, OAW site reported the first confirmed case of measles in an Afghan evacuee; during the subsequent 10 days, five additional cases were identified across multiple sites (*1*). On September 6, OAW response leadership learned that 16 evacuees at Joint Base McGuire-Dix-Lakehurst (JBMDL) had been exposed to a patient with confirmed measles during a September 3 United States-bound flight. Because of low routine measles vaccination coverage rates in Afghanistan (*2*), risk for measles transmission was high among evacuees at JBMDL, a population that would expand to >10,000 persons living in large tents and multifamily rooms, if any exposed evacuees developed measles. During September 7–9, the JBMDL OAW public health team, with support from local and state health departments and guidance from CDC, provided measles, mumps, and rubella (MMR) vaccine or immunoglobulin to exposed persons. Because of delayed reporting of the exposures and challenges locating evacuees, whose lodgings assignments were not always well documented or might have changed, postexposure prophylaxis was not administered within the recommended time frame.* Exposed persons were asked to quarantine and complied; however, because of space constraints, they were not moved into quarantine until 1 week after the exposure. None of the evacuees exposed to the patient on September 3 experienced measles signs or symptoms^†^ during quarantine. This activity was reviewed by CDC and was conducted consistent with applicable federal law and CDC policy.^§^

Although MMR vaccination was already part of the immigration medical exam (*3*), the planned rate of 250 exams per day would have been inadequate to mitigate a potential measles outbreak, in light of the large number of evacuees housed at JBMDL. The public health team, in coordination with OAW leadership, recommended an immediate mass MMR vaccination campaign for all evacuees. The campaign was part of a coordinated effort to vaccinate evacuees at U.S. OAW sites and overseas transit locations after measles cases had been identified. Immigration medical exams were paused, and personnel were reassigned for campaign activities. The campaign ran during September 8–12; a total of 8,849 of 9,503 evacuees (93%) were screened for eligibility and a reliable record of vaccination. Vaccines were administered to eligible persons without documentation of previous vaccination, according to CDC recommendations.^¶^ Age-specific vaccination rates were calculated by merging vaccination and registration data. By September 12, among the 9,065 eligible evacuees residing at JBMDL, 7,962 (88%) had been vaccinated (Table). After the vaccination campaign, immigration medical exams resumed and were expected to identify any remaining unvaccinated persons. By October 31, among a total of 12,670 eligible evacuees, 12,437 (98%) had received the MMR vaccine.**

**TABLE:** Selected characteristics and measles, mumps, and rubella vaccination rates among Afghan evacuees — Joint Base McGuire-Dix-Lakehurst, New Jersey, September 12 and October 31, 2021

Characteristic	Total and vaccinated JBMDL evacuee population on specified date*			
	September 12		October 31	
	Total population	Vaccinated^†^ no. (%)	Total population	Vaccinated^§^ no. (%)
**Total eligible^¶^**	**9,065**	**7,962 (88)**	**12,670**	**12,437 (98)**
**Age group upon arrival**				
6 mos–4 yrs	**1,396**	1,192 (85)	**1,925**	1,907 (99)
5–19 yrs	**3,085**	2,712 (88)	**4,213**	4,175 (99)
20–49 yrs	**4,191**	3,708 (88)	**5,952**	5,813 (92)
≥50 yrs	**389**	348 (89)	**560**	534 (95)
Unknown	**4**	2 (50)	**20**	8 (40)
**Sex**				
Female	**3,729**	3,275 (88)	**5,152**	5,051 (98)
Male	**5,330**	4,683 (88)	**7,430**	7,315 (98)
Unknown	**6**	4 (67)	**88**	71 (81)

**Abbreviations:** JBMDL = Joint Base McGuire-Dix-Lakehurst; MMR = measles, mumps, and rubella.

* Cumulative population. Departures from the base have not been subtracted.

^†^ Includes persons who were vaccinated while at JBMDL and 40 persons who provided reliable proof of MMR vaccination.

^§^ Includes persons who were vaccinated while at JBMDL and 57 persons who provided reliable proof of MMR vaccination.

^¶^ Persons eligible for MMR vaccination include nonpregnant persons aged ≥6 mos.

The public health team reinforced measles surveillance by briefing OAW medical providers on the measles case definition and reporting procedures. In September, four suspected measles cases were identified at JBMDL; all received negative test results^††^ from the New Jersey Department of Health. On October 3, a male infant aged 4 months was evaluated at the OAW medical facility with fever, diaper rash, and diarrhea; a fine maculopapular rash was observed on the chest but not the face. The infant was transferred to a local pediatric hospital and received a positive measles test result on October 6. The infant was not a known contact and was not age-eligible for MMR vaccination during the mass vaccination campaign; all other members of his family had been vaccinated on September 10. The infant arrived on September 9 with rash onset on October 3, indicating at least one undetected primary case at JBMDL.

Contact tracing identified 56 unvaccinated persons who lived in the same residential tent or were registered at the OAW medical facility on the same days as the infant with measles. Among these persons, 54 (96%) were not vaccine-eligible because of age (18; 32%), pregnancy (35; 63%), or medical contraindication to vaccination (one; 2%) at the time of the campaign. The public health team located and moved 45 (83%) of these persons into quarantine (11 persons could not be located). Among the 45 persons who were located, MMR vaccine was administered to the six eligible persons; immunoglobulin was offered to the 39 who remained ineligible, 36 (92%) of whom received it.^§§^ No one developed measles symptoms during quarantine; as of February 1, 2022, no additional cases had been reported.

Early mass vaccination and other response efforts successfully halted the spread of measles; however, inaccurate documentation of evacuee lodging locations led to delays in locating or the inability to locate some exposed persons, and insufficient space resulted in delaying quarantine of exposed persons. Also, reliance on off-site laboratory testing delayed confirmation of suspected cases and prevented serologic testing of pregnant women, which would have eliminated the need to administer immunoglobulin to immune women. Future missions might consider vaccination of persons before arrival, reinforcing disease surveillance, and expanding capabilities for isolation, quarantine, and local testing.

## Footnotes

* For postexposure prophylaxis, MMR vaccine should be administered within 72 hours or immunoglobulin within 6 days of initial measles exposure. All 16 exposed persons were placed in a 21-day quarantine based on date of last exposure (i.e., September 3). https://www.cdc.gov/measles/hcp/index.html#prophylaxis; https://www.cdc.gov/vaccines/pubs/surv-manual/chpt07-measles.pdf

† Quarantined persons were monitored for signs and symptoms of measles per the case definition of the Council of State and Territorial Epidemiologists. https://ndc.services.cdc.gov/case-definitions/measles-2013/

§ 45 C.F.R. part 46, 21 C.F.R. part 56; 42 U.S.C. Sect. 241(d); 5 U.S.C. Sect. 552a; 44 U.S.C. Sect. 3501 et seq.

¶ Infants aged <6 months and pregnant women were not eligible to receive MMR vaccine. Varicella vaccine was offered to all nonpregnant persons aged ≥12 months, and COVID-19 vaccines were offered to all persons aged ≥12 years, consistent with guidance at the time. https://www.cdc.gov/vaccines/schedules/hcp/imz/child-adolescent.html; https://www.cdc.gov/vaccines/schedules/hcp/imz/adult.html

** Date of arrival was not available for 609 evacuees; they were included in the October 31 data timepoint.

†† Persons with suspected cases received reverse transcription–polymerase chain reaction testing for the presence of measles virus RNA.

§§ Eleven of 13 infants, 24 of 25 pregnant women, and one woman who had medical contraindications for MMR vaccine received immunoglobulin. Two additional pregnant women received immunoglobulin but were subsequently unable to be located and moved into quarantine.
